# Inventory management practices: implications on the pharmaceuticals expenditure of rabies vaccine in public health facilities, Namibia

**DOI:** 10.1186/s12913-023-09790-0

**Published:** 2023-08-02

**Authors:** Alemnew Dessie Shibabaw, Hilma N. Nakambale, Varsha Bangalee

**Affiliations:** 1 School of Health Sciences, Department of Pharmaceutical Sciences, University of KwaZulu-Natal, Westville Campus, Private Bag X54001, Durban, 4000 South Africa; 2 Ministry of Health and Social Services, Windhoek, Namibia

**Keywords:** Rabies vaccine, Inventory management, Pharmaceutical expenditure

## Abstract

**Background:**

To achieve well-regulated distribution, storage, and utilization of the rabies vaccine, health facilities should adhere to standard operating procedures. In Namibia, information on inventory management, utilization, monitoring, and reporting of rabies vaccine adherence to standard operating procedures in public healthcare facilities is insufficient. The aim of this study was to assess adherence to rabies vaccine standard operating procedures and inventory management and to compare rabies vaccine expenditure to the number of patients who received rabies vaccination at the Ministry of Health and Social Services’ public healthcare facilities from 2018 to 2020.

**Methods:**

A cross-sectional, web-based questionnaire consisting of closed-ended questions was sent to 147 pharmacy staff and warehouse managers working in the 14 regions of Namibia during the period of May 1, 2021, to June 2, 2021. The overall expenditure and the total number of patients vaccinated from 2018 to 2020 were obtained from national-level logistic and vaccination program coordinators. Data were coded and transcribed into Microsoft® Excel® 2013 and analyzed using SPSS® version 27.

**Results:**

One hundred and thirty-three completed questionnaires were received from sixty-nine public health centers and hospitals. The group of respondents consisted of pharmacist assistants (50%), pharmacy technicians (12%), pharmacists (36.8%), senior pharmacists (0.8%), and chief pharmacists (1.5%). Overall, adherence to standard operating procedures was poor (27.1%). Rabies vaccine distributed to public health facilities from 2018 to 2020 was worth N$75,381,419.91 (~ US$4,074,671.46) and was expected to vaccinate 87,269 patients; however, only 95 cases of both rabies and rabid dog-bite patients were reported. The major inventory management challenges for public healthcare facilities include an inadequate number of pharmacy staff, poor adherence to standardized pharmaceutical warehousing, lack of regular supervision, and inadequate staff training.

**Conclusion:**

Inventory management practices in public healthcare facilities were not in compliance with standard operating procedures. There is a significant discrepancy between rabies vaccine expenditure and the number of patients that were vaccinated. Therefore, there is a need for adequate staff training on inventory management and regular facility supervision to enforce optimal rabies vaccine inventory management practices.

**Supplementary Information:**

The online version contains supplementary material available at 10.1186/s12913-023-09790-0.

## Background

Effective pharmaceutical inventory management is crucial in ensuring that vaccines are continuously available in the right quantities and quality at every stage of the vaccine supply chain [1]. The Ministry of Health and Social Services (MoHSS) in Namibia has implemented a Standard Operating Procedures (SOP) manual for inventory management of vaccines and other pharmaceutical commodities in healthcare facilities. The SOP manual was designed to standardize stock management practices in public healthcare facilities. The manual further classifies certain classes of pharmaceuticals as security items, including the rabies vaccine, which takes up a significant portion of the annual pharmaceutical budget [2] and remains highly susceptible to theft. The Central Medical Store (CMS) – which is the division of the MoHSS responsible for acquiring and distributing pharmaceutical commodities to respective regional stores and hospitals – has additionally implemented an integrated pharmaceutical logistics system (IPLS) to ensure an effective pharmaceutical supply chain [3].

The World Health Organization (WHO) estimates that more than 59,000 rabies-related deaths occur globally every year, and over 95% of those occur in Asia and Africa [4]. Rabies is a neglected zoonotic disease [5], and up to 99% of human cases are dog mediated. Thus, the most effective measure for rabies control is ensuring high vaccination coverage among the dog population [4]. Rabies can also be prevented in humans through timely and appropriate administration of post-exposure prophylaxis (PEP), which involves immediately administering the rabies vaccine or human or horse-derived rabies immune globulin (HRIG) after a dog bite or other exposures. It has been estimated that without PEP, approximately three million people worldwide would die every year from rabies [5]. Rabies is regarded as a notifiable disease in Namibia according to the Animal Health Act 1 of 2011^6^. Between 2011 and 2017, 113 human rabies cases were reported in Namibia, mainly from the Northern Communal Areas [6]. Recently, there have been several media reports alluding to low stock levels of medicines at state hospital pharmacies, as well as incidences of medicine theft, including rabies vaccine, which was confiscated by the police while being smuggled across the border [7].

To address poor inventory management, theft, and other challenges, Namibia has implemented several measures to build a strong pharmaceutical inventory management system to monitor stock. At the healthcare facility level, these measures include the use of systems and tools such as the National Pharmaceutical Dashboard and the Facility Electronic Stock Card (FESC) [8]. The FESC which was launched in 2016, is now being used in all health centers, district, intermediate and central hospitals in Namibia to manage all pharmaceutical supplies including rabies vaccine. Furthermore, there are significant challenges among healthcare workers regarding following standardized practices and appropriate documentation of pharmaceutical transactions to generate relevant reports [9]. To address these challenges, Management Sciences for Health (MSH), in collaboration with the United States Agency for International Development (USAID), assisted the MoHSS in implementing an inventory management system in public health facilities to strengthen the supply chain for medicines [10].

Poor inventory management practices and limited usage of pharmaceutical information system data for assessing medicine management activities have been reported at both the national and healthcare facility levels in Namibia [9]. This study assessed adherence to rabies vaccine SOPs for inventory management activities and determined the expenditure on the rabies vaccine in comparison to the number of patients who received rabies vaccination at the MoHSS public healthcare facilities. The study provides an empirical snapshot of the current rabies vaccine inventory management practices and baseline information to track progress and improvement in rabies vaccine stock management over time [11].

## Methods

### Study design

This study was a cross-sectional, quantitative, multicenter descriptive study conducted across the 14 regions in all public hospitals and health centers (37 health centers, 27 district hospitals, four intermediate hospitals, and one national central hospital). In this study, pharmacy staff and warehouse managers employed as pharmacy professionals at MoHSS were purposively selected as the study population. The selection of respondents was based on the assumption that pharmacy professionals have skills in using pharmaceutical tools for proper pharmaceutical inventory management. Consequently, the study excluded any nurses involved in pharmaceutical inventory management activities.

### Data collection

Two types of questionnaires were administered in English, one for the national-level logistic and vaccination coordinators and the other for pharmacy heads and warehouse managers. The questionnaire for facility-level staff was structured into six sections consisting of 31 questions. Semi-structured questionnaires were used to collect information on the facility and pharmacy professionals’ background, ordering systems for rabies vaccine by facilities, CMS delivery of rabies vaccine to the facility, storage security, factors influencing inventory management practices, expenditure, monitoring and evaluation activities of rabies vaccine stock movement of the facility under study, adapted from the SOP of MoHSS. The questionnaire for national-level staff was structured into two sections with a total of 11 questions to collect information on the expenditure of rabies vaccine distributed to public health facilities at the national level and the number of clients vaccinated and reported to the national health information system (NHIS). The national-level logistic and vaccination program coordinators completed the questionnaire to obtain the aggregated overall expenditure and the total number of patients vaccinated in the country from 2018 to 2020. The questionnaires were piloted with two pharmacists from the target populations who were not selected as participants to ensure their reliability and appropriateness. The questionnaire was amended following the recommended revisions. The questionnaires were distributed electronically via email to 147 participants between May 1, 2021, and, June 2, 2021.

### Sampling

The number of participants included in the study was calculated using GPower software version 3.1.9.4. An estimated sample size of at least 133 participants was required to detect an effect size of 0.3 about 80% of the time at four degrees of freedom. Therefore, the required minimum sample size was 133. The expected non-response rate was 10%, thus the maximum sample size was 147 participants. The number of participants per region was determined based on the number of health facilities in each region under study. Sampling without replacement was used to draw a sample. All the names of participants per region were put in a cylindrical transparent box and drawn lottery-wise until all allocated sample participants were drawn. The national-level logistic and vaccination program coordinators were included in the study to determine the overall expenditure and the total number of patients vaccinated in the country.

### Data analysis

The data collected from the web-based survey was exported into a Microsoft Excel spreadsheet, and was coded and analyzed accordingly using the Statistical Package for Social Science (SPSS) version 27. Descriptive and inferential statistical analysis methods were used to understand and derive conclusions from the collected data. Categorical variables were described as counts and percentage frequencies, and simple bar charts were used to visually display categorical variables. Chi-square and Fisher’s exact tests (for small frequencies) were used to determine the associations between categorical variables. Factors associated with the effectiveness of inventory management practices were determined using logistic regression (univariate and multiple). Stepwise regression was used to determine the most important factors for effective inventory management. All the inferential statistical analysis tests were conducted at a 5% level of significance.

## Results

### Overview of respondent’s demographics

In total, 133 questionnaires were completed (response rate of 90.5%). The respondents comprised 57.9% females. Pharmacist assistants completed (n = 65, 48.9%) questionnaires, followed by pharmacists (n = 49, 36.8%), pharmacy technicians (n = 16, 12.0%), chief pharmacists (n = 2, 1.5%), and a senior pharmacist (n = 1, 0.8%). The Kavango East and Omusati regions accounted for (n = 14, 10.5% for both regions), while the Omaheke region represented the smallest number of participants (n = 3, 2.3%) (Table 1). Educational qualifications attained by the respondents were as follows: college certificates (n = 61, 45.9%); diplomas (n = 19, 14.3%); bachelor’s degrees (n = 42, 31.6%); and postgraduate degrees (n = 11, 8.3%). There was an equal number of respondents who had less than 5 years of experience and between 6 and 10 years of experience in practice (n = 53, 39.8% for both groups). This was followed by those who had practice experience between 11 and 15 years (n = 20, 15.0%), and lastly, respondents who had above 16 years of practice experience (n = 7, 5.3%).

**Table 1:** Number of respondents per health facility type and region

Region		Health Facility type				Total
		Health Centre	District Hospital	Intermediate Hospital	Central Hospital	
	Erongo	1	8	0	0	9(6.8%)
	Hardap	4	6	0	0	10(7.5%)
	Karas	3	7	0	0	10(7.5%)
	Kavango East	1	8	5	0	14(10.5%)
	Kavango West	2	7	0	0	9(6.8%)
	Khomas	4	0	5	2	11(8.3%)
	Kunene	3	5	0	0	8(6.0%)
	Ohangwena	3	5	0	0	8(6.0%)
	Omaheke	0	3	0	0	3(2.3%)
	Omusati	2	12	0	0	14(10.5%)
	Oshana	4	1	5	0	10(7.5%)
	Oshikoto	2	4	3	0	9(6.8%)
	Otjozondjupa	2	7	0	0	9(6.8%)
	Zambezi	2	6	1	0	9(6.8%)
**Total**		33(24.8%)	79(59.4%)	19(14.3%)	2(1.5%)	133(100%)

### Health facilities background information and pharmacy staff

More than half of the respondents (n = 74, 56%) indicated that they had a secured warehouse in compliance with the SOPs. Most of the respondents (n = 111, 83.3%) reported that pharmaceutical warehouses in public healthcare facilities were managed by a pharmacy professional (Table 2). Only 48 respondents (36.1%) reported that they had received training on medicine inventory supply chain management in the last 5 years.

Regarding the adequacy of pharmacy staff, (n = 94, 70.7%) respondents stated that pharmacy providers were not sufficient to effectively offer services in the healthcare facilities. Most respondents reported that they had individual staff members purposely assigned to manage inventory and warehousing activities (n = 111, 83.5%). However, 108 (81.2%) respondents reported not having dedicated supportive staff/data clerks to assist in PMIS for pharmacy services.

**Table 2:** Overview of warehouse security for rabies vaccine storage, warehouse management by pharmacy professionals, and availability of support staff/data clerks to assist PMIS.

Region		Secured warehouse in compliance with SOP		Warehouse managed by pharmacy professional		Support staff/data clerk for recording and reporting PMIS	
		Yes	No	Yes	No	Yes	No
	Erongo	3	6	7	2	0	9
	Hardap	6	4	7	3	2	8
	Karas	7	3	7	3	1	9
	Kavango East	11	3	9	5	3	11
	Kavango West	6	3	6	3	0	9
	Khomas	6	5	10	1	9	2
	Kunene	5	3	7	1	1	7
	Ohangwena	2	6	7	1	1	7
	Omaheke	0	3	3	0	0	3
	Omusati	7	7	14	0	3	11
	Oshana	6	4	9	1	1	9
	Oshikoto	5	4	9	0	0	9
	Otjozondjupa	6	3	7	2	1	8
	Zambezi	4	5	9	0	3	6
**Total**		74(55.6%)	59(44.4%)	111(83.5%)	22(16.5%)	25(18.8%)	108(81.2%)

### Ordering and distribution of rabies vaccine

Most respondents (n = 126, 94.7%) used consumption data from stock cards and Facility Electronic Stock Card (FESC) to quantify and place rabies vaccine orders from CMS, while (n = 5, 3.8%) reported that they used both morbidity data and consumption data from stock cards/FESC (Table 3). Most respondents (n = 111, 83.3%) were satisfied with the rabies vaccine stock quantity received by their facilities from CMS. Among the respondents who did not receive sufficient quantities of rabies vaccine, 17 (out of 22) encountered stockouts (Table 3). Regarding proper documentation (e.g., delivery books, delivery notes, and invoices) required for managing stock, 51.9% (n = 69) of the respondents stated that CMS made deliveries with proper documentation, while 44.4% stated that the stock was sometimes delivered without proper documentation (Table 3). While regarding accountability for receiving stock, (n = 95, 71.4%) of the respondents reported that there was proper handover.

**Table 3:** Key practices and experiences for rabies vaccine ordering and delivery from supplier- CMS by health facility type

Practices and experiences		Health Facility Type				Total
		Health Center	District Hospital	Intermediate Hospital	Central Hospital	
Baseline data used in ordering rabies vaccine from CMS	Morbidity Data	1	1	0	0	2(1.5%)
	Consumption	31	74	19	2	126(94.7%)
	Both Methods	1	4	0	0	5(3.8%)
Satisfied with the quantity received from CMS	Yes	28	67	14	2	111(83.5%)
	No	5	12	5	0	22(16.5%)
Encountered any stock out of rabies vaccine	Yes	4	11	4	0	17(12.8%)
	No	1	1	1	0	3(2.3%)
CMS-delivery of rabies vaccine with-documents	Yes	14	44	9	2	69(51.9%)
	No	1	3	1	0	5(3.8%)
	Sometimes	18	32	9	0	59(44.4%)
Handover of rabies vaccine by CMS	Yes	24	56	13	2	95(71.4%)
	No	9	23	6	0	38(28.6%)

### Respondents’ knowledge and perceptions on factors affecting rabies vaccine inventory management

A total of 44 respondents (33.1%) indicated that they were not aware that rabies vaccine is regarded as a security item. Most of the respondents (n = 117, 88%) agreed that a lockable and secure storage area had a positive impact on improving rabies vaccine stock inventory management. Furthermore, (n = 101, 75.9%) of the respondents agreed that a warehouse managed by one responsible person maximizes the efficiency of inventory management and adherence to SOPs. Most respondents (n = 122, 91.7%) agreed that having adequately trained staff in a facility improves inventory management (Table 4).

A total of 105 respondents, comprising 79% of the participants, agreed that the pharmacy head should approve all stock transactions in the facility. A significant portion of the respondents (n = 125, 94%) agreed that capturing any transactions on FESC in a timely manner maximizes accountability and transparency. Moreover, to monitor rabies vaccine usage in the outpatient department, (n = 51, 38.6%) of the respondents agreed that a list of rabies PEP vaccinated patients should be submitted to replenish stock, while 42.1% of respondents agreed that returning all used vaccine vials should be a prerequisite for replenishing stock from the main pharmacy (Table 4).

Approximately 74.4% of the respondents (n = 99) concurred that conducting weekly/monthly reconciliations of distribution data from the pharmacy and the vaccine administering department is crucial for effective inventory stock management. In addition, (n = 87, 65.4%) of the respondents agreed that having standardized guidelines for registering patients for rabies vaccination can have a positive impact on rabies vaccine stock management, and 74.4% (n = 99) agreed that annual auditing for any transaction can maximize accountability and transparency across the system.

**Table 4:** Respondents level of agreement on key factors affecting rabies vaccine inventory management by facility level

Key factors		Health Facility Type				Total (Percentage)
		Health Center	District Hospital	Intermediate Hospital	Central Hospital	
Storage area should only be accessible by one pharmacy professional	Agree	25	62	14	0	101(76%)
	Neutral	7	11	4	2	24(18%)
	Disagree	1	6	1	0	8(6%)
Adequately trained staff on inventory management is important for rabies inventory management	Agree	30	74	16	2	76(91.7%)
	Neutral	3	1	2	0	6(4.5%)
	Disagree	0	4	1	0	5(3.8%)
Pharmacy head approval should be required	Agree	28	64	13	0	105(79.0%)
	Neutral	4	13	5	2	24(18.0%)
	Disagree	1	2	1	0	4(3.0%)
Transactions should always be captured on FESC	Agree	31	74	18	2	125(94%)
	Neutral	2	4	1	0	7(5.3%)
	Disagree	0	1	0	0	1(0.8%)
Rabies vaccine vaccinated patients list should be a prerequisite for ordering	Agree	13	31	7	0	51(38.6%)
	Neutral	14	31	9	1	55(41.1%)
	Disagree	6	17	3	1	27(20.3%)
Getting back used rabies vaccine vials should be a prerequisite for ordering	Agree	14	31	10	1	56(42.1%)
	Neutral	12	30	7	0	49(36.8%)
	Disagree	7	18	2	1	28(21.1%)
Annual auditing for transaction should be mandatory	Agree	27	60	12	0	99(74.4%)
	Neutral	5	14	4	2	25(18.8%)
	Disagree	1	5	3	0	9(6.8%)
Always track transaction of rabies vaccine	Agree	29	60	10	0	99(74.5%)
	Neutral	3	10	7	2	22(16.5%)
	Disagree	1	9	2	0	12(9.0%)
Standardized guidelines for immunization should be used at all times	Agree	23	49	14	1	87(65.4%)
	Neutral	9	23	4	1	37(27.8%)
	Disagree	1	7	1	0	9(6.8%)

### Factors influencing rabies vaccine inventory management

Respondents reported that the following would positively influence inventory management: adequate training on inventory management (n = 98, 73.7%); availability of trained/skilled staff to effectively use computerized stock management (n = 97, 72.9%); presence of a drug therapeutic committee (n = 86, 64.7%); supportive supervision and on-the-job training (n = 85, 63.9%); regular monitoring of inventory levels (n = 80, 60.2%); adequate personnel (n = 71, 53.4%); sufficient warehouse space (n = 49, 36.8%); and lastly, availability of manual/SOPs at the facility (n = 46, 34.6%) (Table 5).

**Table 5:** Factors influencing the effectiveness of inventory management to adhere SOPs.

Factors for the effectiveness of inventory management	Frequency (Percentage)
Adequate training on inventory management and use	98(73.7%)
Trained staff to use effectively computerized stock management	97(72.9%)
Functional drug therapeutic committee	86(64.7%)
On-time supervision and on job training by regional and national staff	85(63.9%)
Inventory levels regularly monitoring	80(60.2%)
Having adequate personnel	71(53.4%)
Having sufficient warehouse space for storage	49(36.8%)
User manual/SOP in place	46(34.6%)

**Note**: N = 133

### Monitoring and evaluation of rabies vaccine inventory management

Regarding the monitoring of stock movement, all participants (n = 133, 100%) reported that they were using either stock cards/FESC in the pharmacy store. A total of 87 respondents (65.4%) reported recording stock on the stock cards/FESC, which shows the stock balance of rabies vaccine on a weekly, monthly, and yearly basis in the pharmacy store. Meanwhile, (n = 68, 51.1%) respondents reported that there was a rabies vaccine register book in use to register patients vaccinated with rabies vaccine in the outpatient department (Table 6).

Forty-six respondents (n = 46, 34.6%) stated that they did not have stock records in the pharmacy store. On SOP compliance, (n = 65, 48.9%) respondents were non-compliant with SOPs as they did not record vaccinated patients in the rabies vaccine register book in the outpatient department. Among those who reported non-compliance to SOPs, 43 respondents stated that inventory management in their facilities was poor. A total of 97 (72.9%) respondents reported that there was no proper documentation system to compare and contrast rabies vaccine expenditure with the number of patients vaccinated (Table 6).

In Namibia, the acceptable range of pharmaceutical wastage rate (% expenditure on expired and damaged stock) is less than 2% at the facility level [12]. In this study, (n = 68, 51.1%) respondents stated that their facilities were within the acceptable range for expired and damaged rabies vaccine stock, while 46% of the respondents indicated that there was no record at all. All respondents indicated that they submitted PMIS reports to the national level, but only 61 out of 133 respondents received feedback from the national level. Among all respondents (n = 96, 72.2%), indicated that there was no regular supervision by the regional pharmacist. Overall, (n = 93, 69.9%) respondents reported that they were satisfied with the level of rabies vaccine inventory management in their health facilities.

**Table 6:** Analysis of rabies vaccine stock record, register of rabies vaccinated patients, and supervision system

Key areas of analysis		Health Facility Type				Total (%)
		Health Center	District Hospital	Intermediate Hospital	Central Hospital	
Tools used for recording of inventory management	Only stock Card	1	1	0	0	2(1.5%)
	Only FESC	6	32	8	2	48(36.1%)
	FESC & Stock Card	26	46	11	0	71(62.4%)
Stock record shows the stock balance regularly	Yes	22	47	16	2	87(65.4%)
	No	11	32	3	0	46(34.6%)
Availability of an outpatient rabies vaccine register book	Yes	17	39	11	1	68(51.1%)
	No	16	40	8	1	65(48.9%)
Expenditure of rabies vaccine and number of patients vaccinated with rabies vaccine	Expenditure tally with patients vaccinated	7	13	9	2	31(23.3%)
	Expenditure > actual use	1	4	0	0	5(3.8%)
	No documentation to compare the usage	25	62	10	0	97(72.9%)
Number of vials-damaged and expired	Acceptable range	18	41	8	1	68(51.1%)
	Not acceptable	2	2	0	0	4(3.0%)
	Not documented	13	36	11	1	61(45.9%)
Regular supervision by regional pharmacist	Yes	10	22	5	0	37(27.8%)
	No	23	57	14	2	96(72.2%)
Overall satisfaction of participants on rabies vaccine inventory management	Good	8	29	3	0	40(69.9%)
	Bad	25	50	16	2	93(30.1%)

### Further analysis to determine significant relationships and differences

To measure the association between categorical variables, chi-square and Fisher’s exact tests (for small frequencies) were used. A statistically significant (p < 0.05) relationship was detected between overall inventory management practice and the following variables: occupation, secured warehouse, baseline-to-order rabies vaccine, vaccine handover, PMIS tools used, stock record, outpatient rabies vaccine patient register, accountability and transparency without patient register, expenditure, number of people vaccinated, PEP vials wastage rate, and regional pharmacist regular supervision.

Multiple logistic regression was conducted to determine the factors associated with the effectiveness of inventory management. A statistically significant difference was noted in the way different respondents responded to certain questions. There was a significant difference (p < 0.05) in overall inventory management practice based on the following variables: professional level of pharmacy cadre, availability of secured warehouse, proper vaccine handover, FESC usage for ordering rabies vaccine, availability of stock record, availability of outpatient rabies vaccine patient register, PEP vials wastage rate, PEP vials wastage documentation system, and active regional pharmacist regular supervision. Furthermore, stepwise regression was used to determine the most influential factors on inventory management. A high PEP vials wastage rate within an acceptable range showed an odds ratio (OR) of 167.33 (95% CI: 2.82-42642.05, p = 0.041) as the most influential determinant factor for poor inventory management.

## Discussion

This study was conducted to ascertain public healthcare facilities’ adherence to pharmaceutical SOPs for inventory management of the rabies vaccine and to compare rabies vaccine expenditure to the number of patients vaccinated with the rabies vaccine in Namibian public healthcare facilities. Adherence to SOPs helps to ensure that there is efficient inventory management practice at all times, clarifies roles and responsibilities, and facilitates the delegation of duties by providing clear and precise instructions [12]. Additionally, SOPs provide standards for monitoring and supervision of pharmacy services and can be used as training tools and a reference document for new staff members [12].

Human resources are a key performance driver for pharmaceutical inventory and supply chain management [13]. Shortage of pharmacy human resources was highlighted by several respondents as a significant challenge that affects the effective implementation of inventory management activities. Similar findings were found in studies from Ethiopia and South Africa, which revealed that medicine management in healthcare facilities is worsened by inadequate availability of experienced pharmacy providers at the facility level [3, 11].

Many factors influence rabies vaccine inventory management and adherence to SOPs. Among these, respondents reported that adequate training on inventory management had the highest positive impact. Pharmacy personnel at different supply chain levels should receive intensive training on inventory management and SOPs to maximize performance in on-time recording of transactions on FESC, placing orders accurately, reducing volumes of expiry, and lowering the rates of stock-outs of pharmaceuticals [3, 9, 11]. A significant number of respondents (72.9%) agreed that the availability of trained staff is an important factor in improving inventory management and SOPs compliance. Most pharmacy staff did not receive adequate training on inventory management at the facility level. For example, some participants did not know that rabies vaccine is a security item. The study also revealed poor handling of rabies vaccine transactions and a lack of accountability and monitoring systems. These challenges could be compounded by inadequate training of pharmacy providers on SOPs and limited inventory management experience among most pharmacy staff with less than five years of experience.

Pharmaceutical supplies need to be stored in a secure and spacious warehouse to enable optimal performance of inventory management activities such as regular stocktaking, inventory reconciliation, first-expired-first-out practices, and traceability of batches [13]. Most of the respondents in the study agreed that having a secure warehouse managed by trained pharmacy professionals has a positive impact on inventory management. The findings of this study underscore the importance of a conducive environment in enabling routine implementation of inventory management activities.

The CMS is responsible for planning and implementing on-time delivery of pharmaceuticals to public healthcare facilities to ensure that the stock demands from all public healthcare facilities are met regularly [14]. The study found that most of the health facilities were utilizing a pull system - receiving rabies vaccine stock according to their demand, although proper documentation was not always done. Using the pull system ensures that healthcare facilities are supplied according to their demand, thus maximizing stock availability and reducing the expiry of vaccines [11].

All pharmaceutical products delivered to the healthcare facilities should be accompanied by appropriate documentation (e.g., delivery books, delivery notes, and invoices) to ensure robust stock movement security and proper handover [15]. Lack of a proper stock handover system by CMS drivers to the facilities was indicated by most respondents. The study confirmed that the current CMS stock delivery handover system is weak due to the incomplete format of the delivery books, as the books only show the total number of boxes for all the items but not individual items as presented in an invoice [3].

Accurate record-keeping is important for inventory management. Health facilities either use stock cards or FESC to record rabies vaccine stock transactions in the pharmacy store. FESC is an electronic PMIS system that automatically calculates health product usage and generates stock status reports, produces an accurate requisition to medical stores for the resupply of health products, and also provides auditable records of transactions for accountability purposes [8]. Rabies vaccine is one of the commodities that are highly vulnerable to theft, due to its high cost. Thus, accurate record-keeping makes it easier to trace and identify missing vials of rabies vaccine. Almost all respondents (94%) agreed that on-time capturing of any transactions on FESC maximizes accountability and transparency. Staff training should emphasize good record-keeping practice.

Regular supervision of pharmaceutical warehousing/distribution operations by national-level staff or regional pharmacists helps ensure that inventory management functions are implemented according to the SOPs [13]. Poor facility supervision and a lack of regular feedback on inventory management from the national level could be important contributing factors to rabies vaccine wastage and mismanagement [11]. Regular supervision is important to identify gaps in inventory management and provide necessary corrective education and training. Facilities also need to be furnished with regular feedback from the national level on inventory management so that they can improve inventory management practices. Therefore, the national level should address challenges in regular facility supervision and provision of feedback to the facilities.

The national-level study respondents reported that there was no focal person for rabies coordination at the national level, even though rabies is a notifiable disease in Namibia. Thus, the rabies control program should be established at the national and regional level or should be integrated into the expanded program on immunization (EPI) unit. Rabies control programs could learn from the EPI, where SOPs for monitoring and reporting systems are in place and enforced at the facility level [4, 16].

The dog-bite data routinely collected at the health facility level and transmitted through the NHIS can improve surveillance data for rabies and provide the basis for budget allocation and monitoring of expenditure for rabies vaccines [17]. The rabies vaccine distributed to all public health facilities from 2018 to 2020 was worth N$75,381,419.91 (~ US$4,074,671.46) and was expected to vaccinate 87,269 patients, which is nine hundred and eighteen times more than what was recorded by NHIS for the 95 cases of both rabies and rabid dog-bite patients reported. While rabies vaccine has a high share of the pharmaceutical budget expenditure at the national level, the estimated annual deaths from rabies in Namibia were only 4 in 2015, with approximately 947 to 6070 PEP administered [18]. Potential cases of misuse and theft would likely explain the high number of rabies vaccines ordered in comparison to the number of rabies cases and deaths estimated. Additionally, poor recording and registration of patients could potentially be a challenge in many facilities, leading to inaccurate monthly and annual estimates. The majority of the respondents (72.9%) confirmed that there was no documentation system to compare and contrast rabies vaccine expenditure with the number of patients vaccinated at the facility level. Similarly, another study done in Namibia has revealed that health providers at the healthcare facility level do not always follow SOPs, nor appropriately document pharmaceutical transactions to generate relevant reports [9]. Standardized guidelines to register patients for rabies vaccination should be in place at each health facility level, and the number of rabies cases and dog-bite patients should be reported on time to the NHIS.

### Limitations

This study solely looked at adherence to SOPs for rabies vaccine inventory management and the national expenditure aggregate for rabies vaccines from all public healthcare facilities. The study did not consider all pharmaceutical supply chain inventory management systems, nor the adherence of clinicians to standard treatment guidelines for vaccine prescribing. Exploration of those factors could provide further insights into rabies vaccine inventory management at the healthcare facility level.

## Conclusion

This study has demonstrated that there are serious deficiencies in rabies vaccine storage security, staff in-service training, and usage of inventory tools to generate relevant reports in most healthcare facilities due to non-adherence to pharmaceutical SOPs. While the results have shown that most healthcare facilities had pharmaceutical inventory management tools in place, most facilities did not have accurate records to generate reports for decision-making. The study revealed that there was a huge gap between rabies vaccine expenditure and the number of patients that were vaccinated and reported at the national level. Therefore, the national supply chain directorate and regional pharmacists need to explore measures to train pharmacy staff on inventory management so that they are better equipped to use and generate relevant on-time reports for decision-making and further improvement. Furthermore, there is a need to establish a management position for the rabies control coordinator at the national level to monitor rabies incidence and vaccine inventory. The public healthcare sector needs to establish measures to ensure rigorous rabies vaccine inventory management practices at all levels of the healthcare system in Namibia.

## Electronic supplementary material

Below is the link to the electronic supplementary material.

Supplementary Material 1

## Data Availability

The datasets used and/or analyzed during the current study are available from the corresponding author on reasonable request.

## List of abbreviations

CMS: Central Medical Store
FESC: Facility Electronic Stock Card
NHIS: National Health Information System
IPLS: Integrated Pharmaceutical Logistics System
MSH: Management Sciences for Health
MoHSS: Ministry of Health and Social Service
PEP: Post Exposure Prophylaxis
PMIS: Pharmaceutical Management Information System
SOP/s: Standard Operating Procedures
SPSS: Statistical Package for Social Science
USAID: United States Agency for International Development
WHO: World Health Organization
