# Supplementary material for: Inventory management practices: implications on the pharmaceuticals expenditure of rabies vaccine in public health facilities, Namibia
Source: BMC Health Serv Res. 2023 Aug 2;23:823. doi: 10.1186/s12913-023-09790-0 (PMC10394911; doi:10.1186/s12913-023-09790-0)
Supplement: Supplementary file 1 — Supplementary Material 1 [file 12913_2023_9790_MOESM1_ESM.docx]

**Annexure S1**

### **Research questionnaire one**

**Questionnaire for pharmacy heads and warehouse manager**

**SECTION A: Demographic Information;** Participant and Health Facility Background information

1. The region where you are working currently? * *Mark only one oval.*

Erongo

Hardap

Karas

Kavango East

Kavango West

Khomas

Kunene

Ohangwena

Omaheke

Omusati

Oshana

Oshikoto

Otjozondjupa

Zambezi

1. Your Health Facility type * *Mark only one oval.*

Health Center

District hospital

Regional Hospital

Intermediate Hospital

Tertiary Health Care-Central Hospital

1. Occupation *

Pharmacist Assistant

Pharmacist Technician

Pharmacist

Senior Pharmacist

Chief pharmacist

1. Gender *

Male

Female

1. Educational Level * *Mark only one oval.*

- Certificate
- Diploma
- Degree
- Postgraduate (honors, masters)
- PHD

1. Years of Working experience * *Mark only one oval.*

- 6-10 years
- 11-15 years
- Above 16 years

**SECTION B: Health facility background Information;** baseline data for staff and infrastructure of the pharmacy at the facility level.

1. Do you have secured and standardized storage for rabies vaccine in your health facility? *

Yes

No

1. Do you have a Pharmaceutical warehouse solely managed by a pharmacy professional? *

Yes

No

1. Do you have support staff/data clerks to assist in logistic management information systems (LMIS) to record or capture all types of pharmaceutical transactions? * *Mark only one oval.*

Yes

No

1. Do you have an adequate number of pharmacy staff in your facility*?

Yes

No

1. Did you receive any pharmaceutical inventory management and quality assurance-related training in the last 5 years? *

Yes

No

1. If your response is "No" to the above questions, is it easily manageable to adhere to SOPs of pharmaceutical inventory management?

Yes

No

**Section C: Ordering system;** in this section, the questions investigate the ordering system from facilities using facility electronic stock Card (FESC) and whether Central Medical Store (CMS) supplied the rabies vaccine with full quantity and deliver the facilities stock with proper documents. This will enable us to find out whether the electronic inventory management system in use is effective and has outputs, as well as the proper handover of the vaccine to the facility, bring positive change to attain Pharmaceutical objectives.

1. Which one are you using as a baseline to order rabies vaccine from CMS in your facility *

Morbidity Data

Consumption data from FESC or stock card

Both methods

Based on funds availability

1. Are you interested in the quantity that you are getting from CMS? *

Yes

No

1. If your response is, "No", did you encounter any stock out
2. of Rabies vaccine in your health facility since Jan 2020?

Yes

No

1. Does CMS deliver Rabies vaccine with proper documentations (Invoices/Crosschecking paper/Picking Slips) *

Yes

No

Sometimes

1. Are you aware that the Rabies vaccine is a 'security item'? *

Yes

No

1. When you are receiving the Rabies vaccine from CMS, is there a proper handover of the vaccine to your facility by CMS drivers using properly designed crosschecking paper? * *Mark only one oval.*

Yes

No

1. Which Pharmaceutical Management Information System (PMIS) tools are you using in your facility to record any transactions while receiving, issuing, or ordering rabies vaccine * *Mark only one oval.*

Stock Card

FESC or Automated Computerized System

Register book

Stock card and FESC

All

**Section D: Factors affecting Rabies vaccine stock inventory management at the facility level;** in this section, the questions investigate the security of rabies vaccine storage area and the stock movement from a facility warehouse to the ward causality or clinics. This will enable us to find out whether the rabies vaccine storage area is accessible by only one staff member and ensuring the use of rabies vaccine to reach the end-user by auditing standard operating procedures.

1. Please provide your level of agreement with the statements below with the factors that influence the effectiveness of the rabies vaccine inventory management system based on your experience. Do you think having-------------- improve the effectiveness of stock management? *


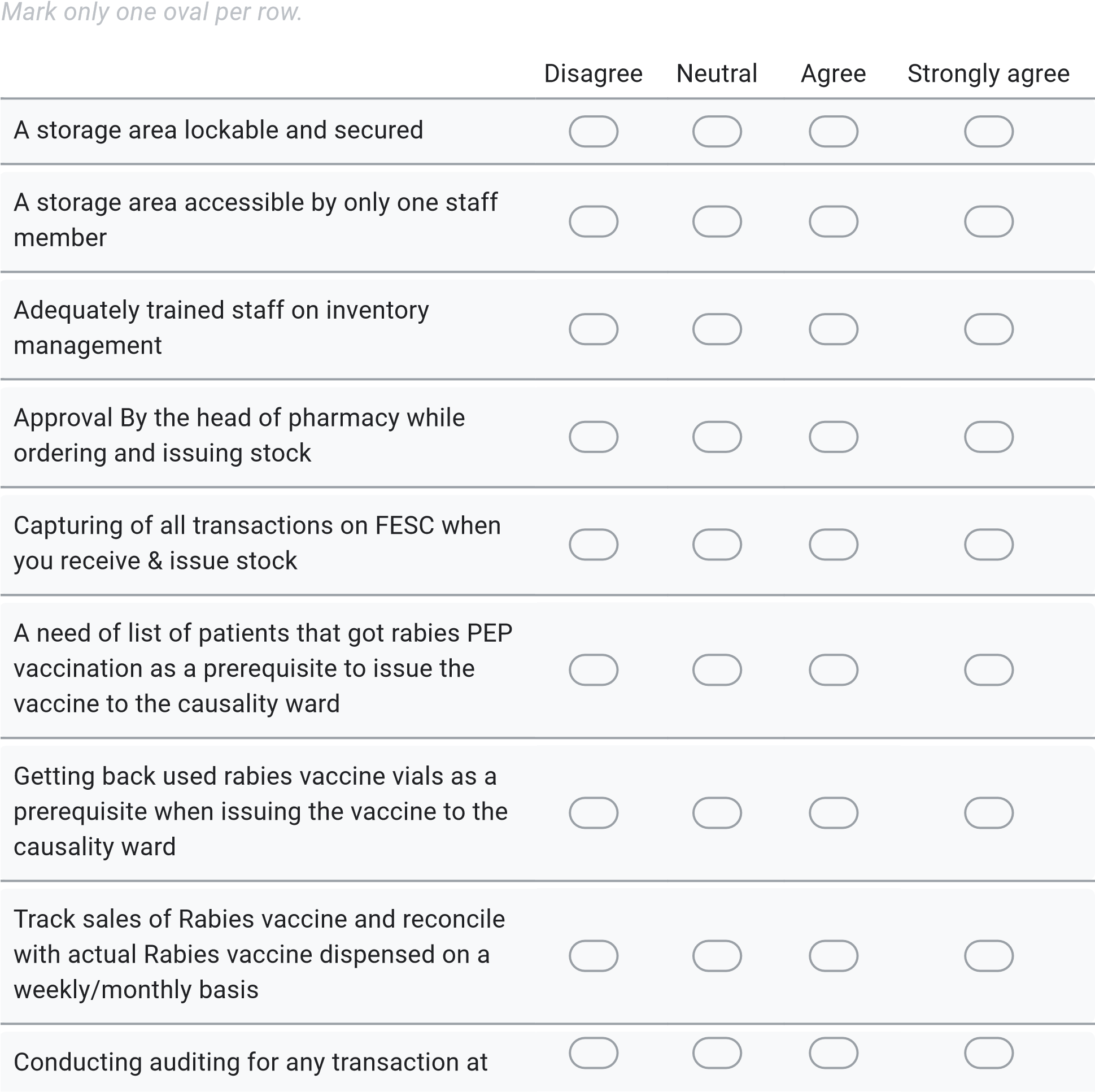


Least once per year

Performing ABC/VEN reconciliation annually

Standardized guideline for rabies vaccine

Immunization like other routine vaccines

1. What are the major factors that improve the effectiveness of rabies vaccine inventory management at your facility? * *Check all that apply.*

- Adequate training on inventory management and its use.
- User manual in place.
- Having a trained Staff to use effectively computerized stock management tools.
- Having adequate personnel.
- Inventory levels regularly monitoring
- On-time supervision and on-job training by regional or national staff
- Having sufficient warehouse space/refrigerator for storage
- Having a functional drug therapeutic committee

**Section E: Monitoring and Evaluation Mechanism;** For monitoring and evaluation of rabies vaccine stock movement at the warehouse and its utilization.

1. What inventory controlling tools are you using at your facility? *

An electronic system (FESC)

Stock card

Stock ledger

All

1. Do you have a stock record either electronically or manually that clearly shows the stock balance of the rabies vaccine on a weekly, monthly, and yearly basis? * *Mark only one oval.*

Yes

No

1. If your response is, "No", how do you evaluate the accountability and transparency with regard to the use of rabies vaccine stock movement to the end-user?

Good

Bad

1. Do you have an outpatient rabies vaccine register with details of the name of the patient, age, sex, biting animal, and category of bite vaccine dose given? *

Yes

No

1. If your response is, "No", how do you evaluate the accountability and transparency of rabies vaccine stock movement to the end-user?

Good

Bad

1. How do you compare the total expenditure of the rabies vaccine dispensed at your facility and the number of people who got a vaccination for PEP in the 2019/2020 financial year? * *Mark only one oval.*

It was in the acceptable range

The expenditure was more than expected compared to the people who got the vaccination

There was no proper documentation system to compare and contrast the usage.

1. How many vials of PEP were wasted last financial year from your facility or how much was the wastage rate? *

On acceptable range according to SOP

Not on acceptable range according to SOP

No any record at all

1. Is there regular supervision of your facility's rabies vaccine inventory management systems by a regional pharmacist? *

Yes

No

1. Are you getting an on-time feedback report after your facility submitted the PMIS indicators report on medicine use to the national-level? * *Mark only one oval.*

- Yes
- No

1. In your opinion based on your responses above, how are the overall Rabies vaccine inventory management, monitoring, and evaluation practices at your facility level? * *Mark only one oval.*

Good

Bad

**Annexure S2**

### **Research questionnaire two**

**Questionnaire for national-level logistic and EPI coordinators**

**SECTION A: Participant background Information**

1. Occupation

----------------------------------------------------------------------------

1. Gender

*Mark only one oval.*

Male

Female

1. Educational level

*Mark only one oval.*

- College Certificate
- Bachelor’s degree
- Postgraduate (honors, masters)
- PHD

1. Years of working experience *Mark only one oval.*
   - Less than 5 years
   - 6-10 years
   - 11-15 years
   - Above 16 years

**Section B: Expenditure of rabies vaccine;** to compare and contrast the annual expenditure of the rabies vaccine with the total population who get vaccinated for PEP and all items distributed to public health facilities.

1. The total quantity of vials and the total value of Rabies vaccine distributed in Namibian dollars to public health facilities across the country from 1 January 2018 to 31 December 2020?

--------------------------------------------------------------------------------------------------------------------------------------------------------------------------------------------------------------------------

1. Do all health facilities report number of patients vaccinated with rabies vaccine to the national-level?

*Mark only one oval.*

Yes

No

1. If "YES", what is the aggregated total number of patients vaccinated with rabies vaccine across the country from 1 January 2018 to 31 December 2020 at the national-level?
2. In VEN analysis, the rabies vaccine is a vital product and it is one of the items from the top ten that was distributed from 1 January 2018 to 31 December 2020 based on ABC analysis?

*Mark only one oval.*

YES

No

1. When you compare the total expenditure of rabies vaccine dispensed and the number of people who got a vaccination for PEP at the national-level in the 2019/2020 financial year.

*Mark only one oval.*

It was in the acceptable range

The expenditure was more than expected compared to the people who got the vaccination

There was no proper documentation system to compare and contrast the usage.

There was no proper reporting channel for the number of patients vaccinated nationally to compare with

1. How do you rate the overall monitoring and evaluation of the service provided by comparing the number of patients vaccinated with the value of rabies vaccine distributed across the country at the national-level?

*Mark only one oval.*

Good

Bad

**Annexure S3**

**Rabies outpatient and inpatient cases in Namibia 2018-2020**

| **Rabies outpatient and inpatient cases** | | | |
| --- | --- | --- | --- |
| **Region** | **Facility Name** | **Number of rabies suspected Cases reported** | **Percentage per facility and region** |
| Erongo Region | Swakopmund Hospital | 3 | 3% |
|  | Walvis Bay Hospital | 2 | 2% |
| **Erongo Region Total** | | **5** | **5%** |
| Hardap Region | Rehoboth Hospital | 1 | 1% |
| **Hardap Region Total** | | **1** | **1%** |
| Karas Region | Keetmanshoop Hospital | 2 | 2% |
| **Karas Region Total** | | **2** | **2%** |
| Kavango Region | Andara Hospital | 1 | 1% |
|  | Rundu Hospital | 17 | 18% |
| **Kavango Region Total** | | **18** | **19%** |
| Khomas Region | Katutura Hospital | 8 | 8% |
|  | Windhoek Central Hospital | 4 | 4% |
| **Khomas Region Total** | | **12** | **13%** |
| Kunene Region | Khorixas Hospital | 4 | 4% |
|  | Opuwo Hospital | 1 | 1% |
|  | Outjo Hospital | 1 | 1% |
| **Kunene Region Total** | | **6** | **6%** |
| Ohangwena Region | Eenhana Hospital | 4 | 4% |
|  | Engela Hospital | 7 | 7% |
|  | Okongo Hospital | 7 | 7% |
| **Ohangwena Region Total** | | **18** | **19%** |
| Omaheke Region | Gobabis Hospital | 1 | 1% |
| **Omaheke Region Total** | | **1** | **1%** |
| Omusati Region | Okahao Hospital | 1 | 1% |
|  | Oshikuku Hospital | 1 | 1% |
|  | Outapi Hospital | 2 | 2% |
|  | Tsandi Hospital | 1 | 1% |
| **Omusati Region Total** | | **5** | **5%** |
| Oshana Region | Oshakati Hospital | 14 | 15% |
| **Oshana Region Total** | | **14** | **15%** |
| Oshikoto Region | Onandjokwe Hospital | 2 | 2% |
|  | Tsumeb Hospital | 1 | 1% |
| **Oshikoto Region Total** | | **3** | **3%** |
| Otjozondjupa Region | Grootfontein Hospital | 5 | 5% |
| **Otjozondjupa Region Total** | | **5** | **5%** |
| Zambezi Region | Katima Mulilo Hospital | 5 | 5% |
| **Zambezi Region Total** | | **5** | **5%** |
| **Grand Total** | | **95** | **100%** |

**Figure S1: Facility electronic stock card (FESC) homepage**

**
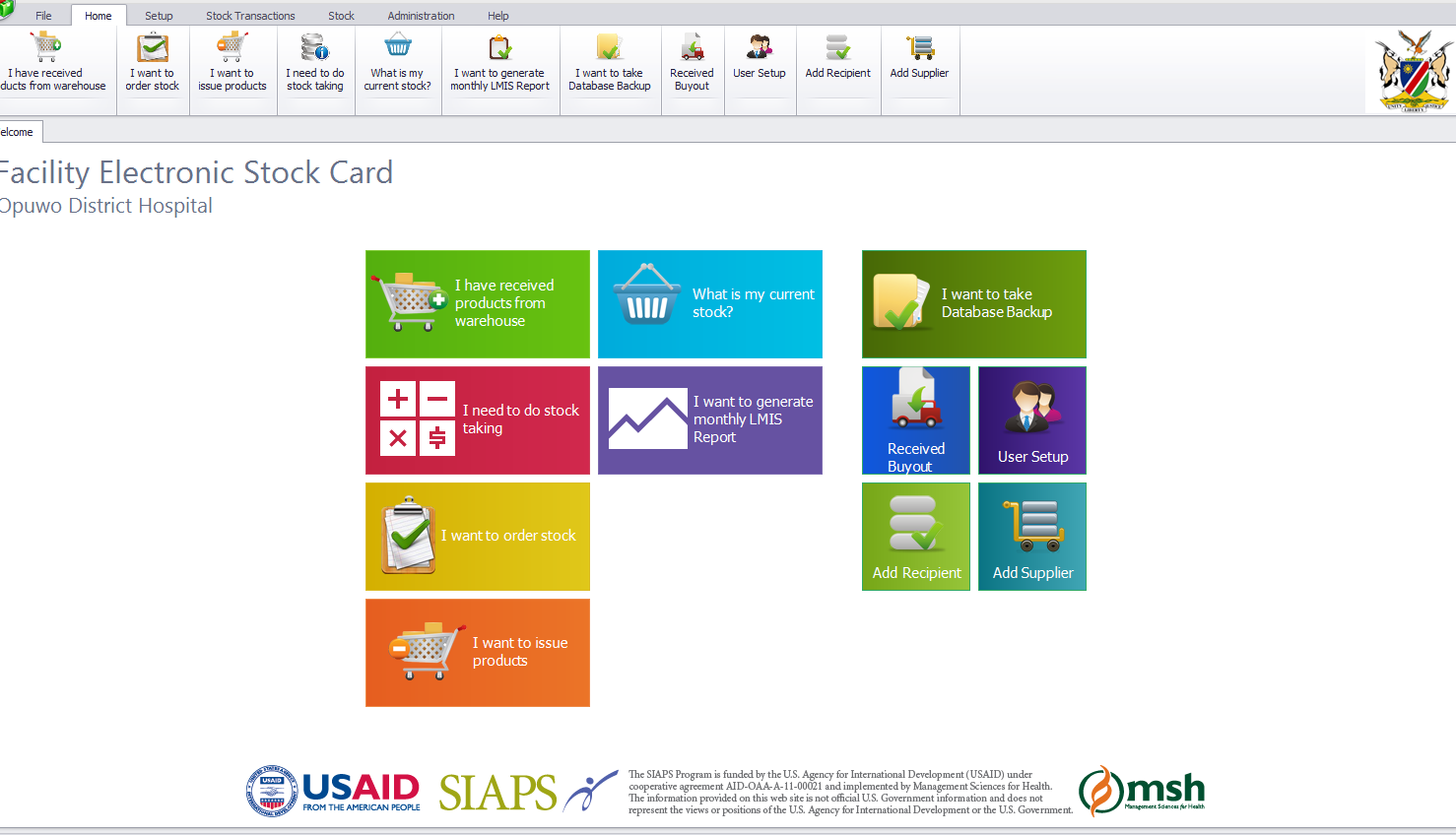
**

*Source: Original*

**Figure S2:  Pharmaceutical Management Information (PMIS) homepage**

**
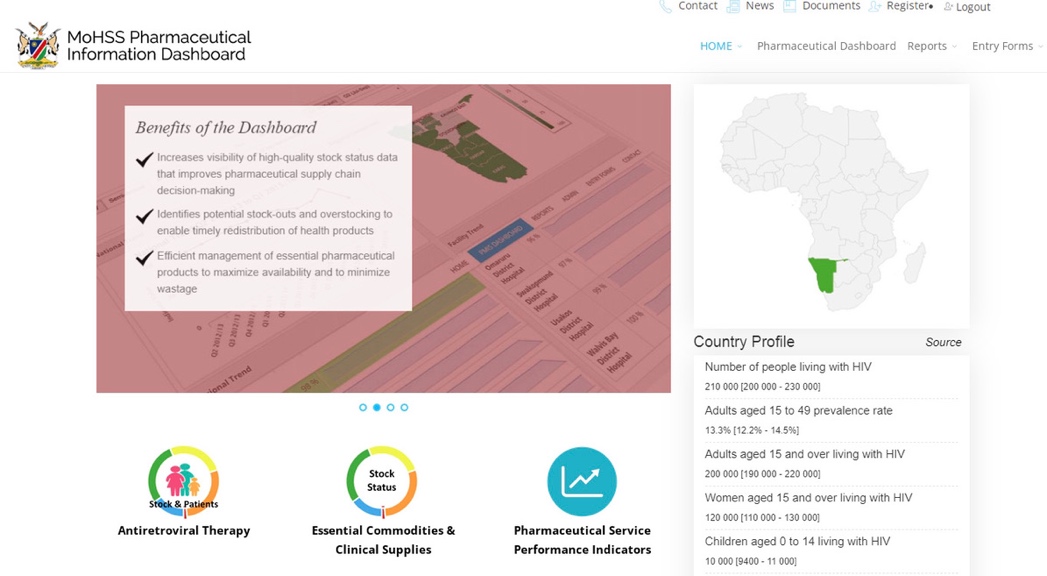
**

*Source: Original*
